# Is bioprosthetic leaflet thrombosis a trigger to valve degeneration?

**DOI:** 10.1136/heartjnl-2017-312861

**Published:** 2018-03-01

**Authors:** Mhairi Katrina Doris, Marc Richard Dweck

**Affiliations:** BHF Centre for Cardiovascular Science, University of Edinburgh, Edinburgh, UK

**Keywords:** aortic stenosis, transcatheter valve interventions

Transcatheter aortic valve implantation (TAVI) has expanded rapidly as a novel therapeutic strategy for patients with severe symptomatic aortic stenosis. This procedure is already well established in patients at intermediate or high risk for conventional surgical valve replacement and, with multiple studies currently evaluating TAVI in low-risk patients, use of this treatment is likely to continue to expand worldwide. While long-term outcome data are lacking, recent results from the Placement of Aortic Transcatheter Valves (PARTNER) trial indicated promising results at 5 years post-implantation, with TAVI valves demonstrating similar haemodynamics to surgical bioprosthetic valves and no evidence of structural valve deterioration on echocardiography.^1^ Despite these promising data, long-term TAVI durability and the mechanisms leading to structural valve degeneration are not fully understood. Improving our understanding of valve durability and the factors leading to TAVI valve deterioration is therefore paramount, particularly as we consider extending this technology to younger patient populations.

In their *Heart* manuscript, Del Trigo *et al* 2 investigate the relationship between the use of anticoagulation therapy and valve haemodynamic deterioration in over 2000 patients post-TAVI. In this cohort, 707 patients were treated with oral anticoagulation therapy with the most common indication being atrial fibrillation. The majority received vitamin K antagonists, with only a small proportion treated with direct oral anticoagulants. At 1 year follow-up, a small but significant increase in mean transvalvular gradient on transthoracic echocardiography was observed in patients not receiving anticoagulation (9.8 mm Hg to 10.3 mm Hg) compared with post-discharge values. By contrast, transvalvular gradients remained stable after 1 year in the patients receiving anticoagulation (9.4 mm Hg and 9.1 mm Hg). Valve haemodynamic deterioration, defined as an increase in transvalvular gradient of ≥10 mm Hg from hospital discharge, had a higher incidence in the group not treated with anticoagulation compared with those receiving anticoagulation (3.7% vs 0.6%, P<0.001). This was observed both in the population as a whole and in a propensity-matched cohort.

This study does have some limitations. The cohort largely comprised patients with well-functioning valves, indeed even those patients diagnosed with valve haemodynamic deterioration had only mild bioprosthetic stenosis, with transvalvular gradients <40 mm Hg. As a consequence, there were too few adverse clinical events in patients with valve haemodynamic deterioration in the matched population (n=7) for meaningful analysis. Another important limitation is that bleeding rates in this cohort were not available, and it is therefore not possible to balance the apparent benefits of anticoagulation on valve durability against the risk of major bleeding. Finally, this is not a randomised controlled trial and there will therefore be both known and unknown confounders that might account for the observed differences.

**Figure 1:**
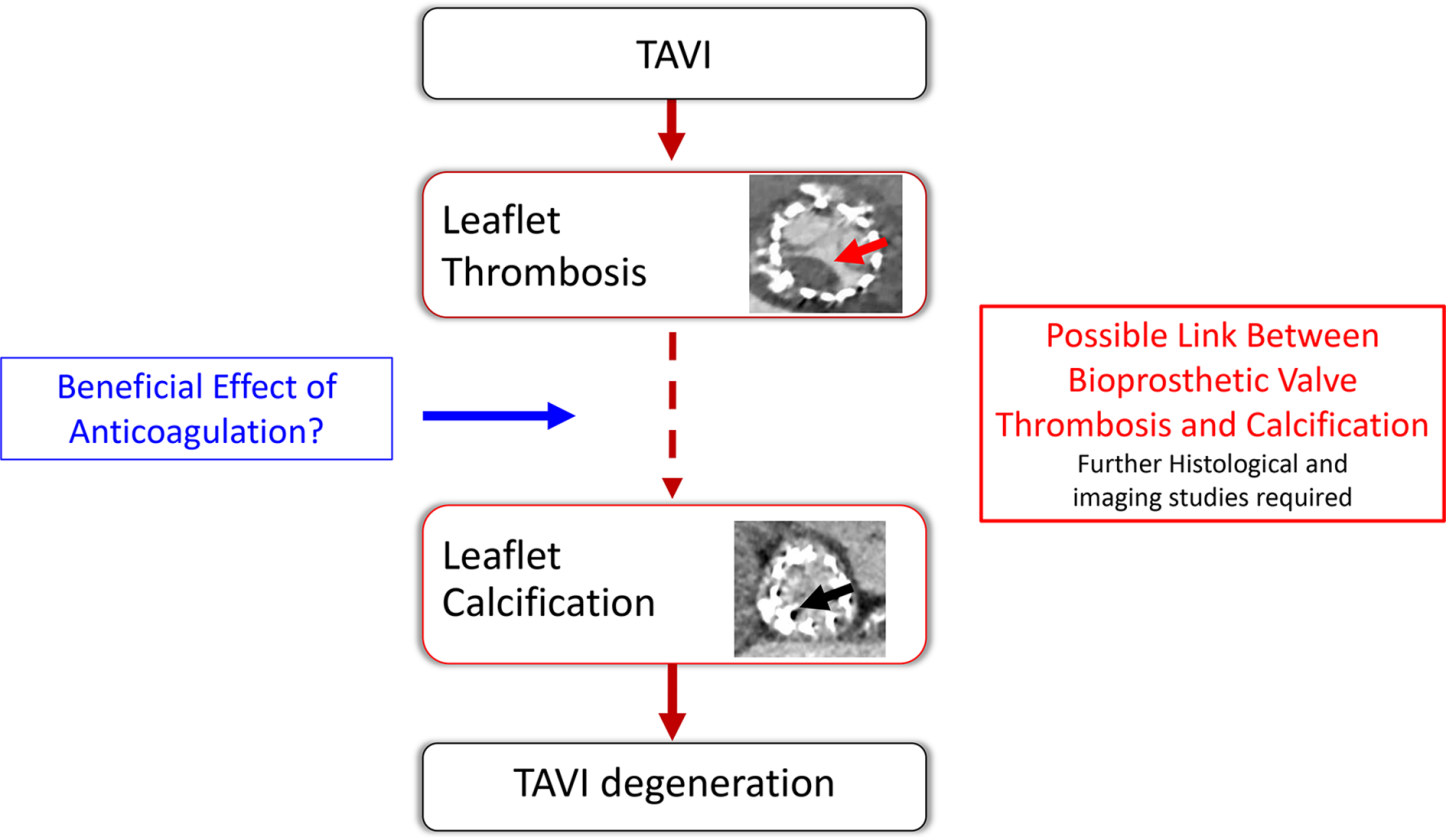
Possible link between bioprosthetic valve thrombosis and calcification. The study by Del Trigo *et al* suggests that anticoagulation may preserve transcatheter aortic valve implantation (TAVI) valve haemodynamics. The potential mechanism for this observation is not clear. Concern has recently grown regarding the incidence of subclinical leaflet thrombosis (red arrow) in patients undergoing TAVI, and it is hypothesised that this may lead to future calcific degeneration of bioprosthetic TAVI valves (black arrow). Future research should focus on investigating this possible mechanistic link between bioprosthetic leaflet thrombosis and valve calcification/degeneration and the role that anticoagulant therapy might play in improving TAVI valve durability.

Nevertheless, the authors are to be congratulated on investigating this important area and highlighting the intriguing hypothesis that anticoagulation therapy post-TAVI may protect against subsequent valve deterioration. This requires a clear mechanistic explanation. Bioprosthetic valve degeneration is predominantly driven by calcification: how might this be linked to antithrombotic therapy? One potential answer is valve leaflet thrombosis. While leaflet thrombosis causing abrupt valve failure occurs in just 1% of TAVI valves, subclinical valve thrombosis has been observed in 7%–10% of implanted TAVI valves.^3 4^ The clinical relevance of this imaging observation has to date been unclear, but perhaps this is not a benign phenomenon, perhaps valve leaflet thrombosis acts as a trigger to calcification and ultimately TAVI valve degeneration (figure 1). This hypothesis is supported by the pathophysiology of native aortic valve stenosis where valve leaflet haemorrhage is associated with more rapid valve calcification and disease progression.^5^ The study by Del Trigo *et al* is therefore important because of the key mechanistic questions raised. In the absence of anticoagulation, do bioprostheses with evidence of leaflet thrombosis calcify and degenerate more quickly? Can histological or imaging studies confirm the association between leaflet thrombosis and early calcification activity? Are surgical bioprostheses also susceptible to similar mechanisms of degeneration? Research must now focus on gaining a greater understanding of the link between valve thrombosis and degeneration, with the hope that ultimately this will help inform optimal management to prolong bioprosthetic valve longevity and improve patient outcomes.
